# A Micro-In-Macro Gastroretentive System for the Delivery of Narrow-Absorption Window Drugs

**DOI:** 10.3390/polym15061385

**Published:** 2023-03-10

**Authors:** Mershen Govender, Thankhoe A. Rants’o, Yahya E. Choonara

**Affiliations:** Wits Advanced Drug Delivery Platform Research Unit, Department of Pharmacy and Pharmacology, School of Therapeutic Sciences, Faculty of Health Sciences, University of the Witwatersrand, 7 York Road, Parktown, Johannesburg 2193, South Africa

**Keywords:** gastroretentive polymer systems, gastrofloatable, micro-in-macro, narrow absorption window drugs, molecular modelling

## Abstract

A micro-in-macro gastroretentive and gastrofloatable drug delivery system (MGDDS), loaded with the model-drug ciprofloxacin, was developed in this study to address the limitations commonly experienced in narrow-absorption window (NAW) drug delivery. The MGDDS, which consists of microparticles loaded in a gastrofloatable macroparticle (gastrosphere) was designed to modify the release of ciprofloxacin, allowing for an increased drug absorption via the gastrointestinal tract. The prepared inner microparticles (1–4 µm) were formed by crosslinking chitosan (CHT) and Eudragit^®^ RL 30D (EUD), with the outer gastrospheres prepared from alginate (ALG), pectin (PEC), poly(acrylic acid) (PAA) and poly(lactic-co-glycolic) acid (PLGA). An experimental design was utilized to optimize the prepared microparticles prior to Fourier Transition Infrared (FTIR) spectroscopy, Scanning Electron Microscopy (SEM) and in vitro drug release studies. Additionally, the in vivo analysis of the MGDDS, employing a Large White Pig model and molecular modeling of the ciprofloxacin-polymer interactions, were performed. The FTIR results determined that the crosslinking of the respective polymers in the microparticle and gastrosphere was achieved, with the SEM analysis detailing the size of the microparticles formed and the porous nature of the MGDDS, which is essential for drug release. The in vivo drug release analysis results further displayed a more controlled ciprofloxacin release profile over 24 h and a greater bioavailability for the MGDDS when compared to the marketed immediate-release ciprofloxacin product. Overall, the developed system successfully delivered ciprofloxacin in a control-release manner and enhanced its absorption, thereby displaying the potential of the system to be used in the delivery of other NAW drugs.

## 1. Introduction

Oral dosing of medicine is the most common and preferred method of administration, although many drugs possess certain characteristics which lead to complex dosing regimens, often resulting in decreased patient compliance [1,2,3,4]. A narrow absorption window (NAW) is such a characteristic and is possessed by many commonly used drugs, including metformin, riboflavin, levodopa and ciprofloxacin. These drugs are primarily absorbed in the proximal area of the small intestine, with minimal to no absorption taking place further down the GIT. This is usually caused by the physicochemical properties of the drugs, such as limited solubility or instability in the alkaline pH of the small intestine [5,6,7]. Drugs with a NAW usually require multiple dosing with very high drug concentrations to achieve therapeutic drug levels, with severe side effects resultantly occurring. For example, the antidiabetic drug metformin, has about only 50% oral bioavailability and a half-life of up to 3 h, necessitating a high dose of 500 mg twice daily or 850 mg once a day. This standard dose can be titrated up to 3 g per day, depending on the patient’s response. The adverse effects resulting from these high doses include diarrhea and anorexia or more serious effects, such as lactic acidosis [8].

Advanced drug delivery systems that prolong the gastric retention of drugs, allowing for controlled release, is therefore a viable option to overcome the concerns of NAW drug delivery. Multiple methods have been investigated to promote gastric retention with the most common approaches being: (i) low density systems, which float on the surface of the gastric fluid; (ii) mucoadhesive systems, which adhere to the lining of the stomach; and (iii) swelling and expanding systems, which increase in size and inhibit passage through the pyloric sphincter. Additionally, a few important design factors must be taken into consideration when formulating a drug delivery system that is intended to have an increased gastric residence time. These include particulate density (to allow for buoyancy in the gastric media), size (units with a diameter of more than 7.5 mm are reported to have a greater gastric-residence time), geometry (tetrahedron and ring-shaped systems have an increased gastric-residence time when compared to other shapes) and the number of units (multi-unit systems are more ideal for gastric retention than single unit systems) [9,10]. For these reasons, multi-unit systems conforming to these requirements are more commonly utilized for gastroretentive drug delivery with these systems being further advantageous in having a greater predictability, a decreased potential of causing localized damage to mucosal linings and a lower risk of dose-dumping [11,12].

**Figure 1:**
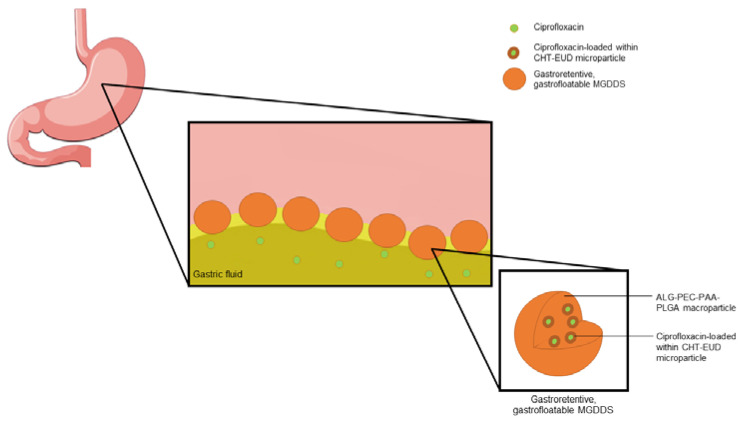
A schematic summarizing the release of the model NAW drug ciprofloxacin from MGDDS.

In the development of such a system, the use of a multi-component platform incorporating microparticles in a macroparticle has the potential to exhibit the ideal characteristics for gastric retention, such as gastro-flotation (flotation in gastric media due to its lower density) and gastro-adherence, in addition to the ideal properties of density, size and shape, as described above [13]. This study therefore provides for the development of a microparticle-in-macroparticle, gastrofloatable and gastroretentive platform for the delivery of NAW drugs (MGDDS). Ciprofloxacin, a broad-spectrum antibiotic with a NAW, has been used as the model-drug for the development of this system [14]. Ciprofloxacin is a weak base and has increased solubility and stability in the acidic environment of the stomach; a common property of NAW drugs [15]. The prepared system functions by retaining the ciprofloxacin-loaded units in the gastric media, slowly releasing the model drug into the gastric contents, thereby allowing for enhanced absorption in the proximal intestine. The MGDDS was developed using chitosan (CHT) and Eudragit^®^ RL 30D (EUD), for their gelling and controlled-release properties, prepared into microparticles, which were thereafter incorporated into an ionic crosslinked gastrosphere for the controlled release of ciprofloxacin over 12 h. The gastrospheres were prepared from alginate (ALG), pectin (PEC), poly(acrylic acid) (PAA) and poly(lactic-co-glycolic) acid (PLGA). ALG and PEC were chosen for their gelling and gastrofloatable properties and their crosslinking potential with calcium ions, with PAA and PLGA utilized for their controlled release, gastroretentive and biodegradation properties [16,17,18,19]. The in vitro characterization of the developed platform was undertaken using FTIR and SEM prior to in vivo evaluation in a Large White Pig model. Additionally, molecular modeling of the drug-polymer interaction was undertaken. A schematic diagram summarizing the gastroretentive and drug release properties of the MGDDS is provided in Figure 1. In this figure, the microparticles containing ciprofloxacin incorporated into the gastrofloatable gastrospheres, as well as the subsequent release of ciprofloxacin into the gastric media is depicted.

## 2. Materials and Methods

### 2.1. Materials

Chitosan (medium molecular weight), ciprofloxacin and sodium tripolyphosphate (TPP) were purchased from Sigma-Aldrich (Sigma-Aldrich Chemie, Steinheim, Germany); alginate (Protanal LF 10/60; Mw 89,000) was purchased from FMC BioPolymer (Drammen, Norway); pectin (Classic CU 701; Mw ≈ 50,000) was purchased from Herbstreith and Fox (Neuenbürg, Germany); poly(acrylic acid) (Carbopol 974P NF; Mw ≈ 3 × 10^9^) was purchased from Noveon (Cleveland, OH, USA); poly(lactic-co-glycolic) acid (Resomer RG 858 S; Mw 190,000–240,000) was purchased from Boehringer Ingelheim (Ingelheim, Germany); and calcium hydroxide was purchased from BDH Chemicals Ltd. (Poole, UK). Eudragit^®^ RL 30D (Mw ≈ 32,000) was received as a gift from Röhm Pharma Polymers (Darmstadt, Germany). All other reagents used were of analytical grade and were employed as purchased, without further purification.

### 2.2. Preparation of the Ciprofloxacin-Loaded Microparticles

The ciprofloxacin-loaded microparticles were formulated by preparing a homogenous solution of ciprofloxacin (50 mg), CHT and EUD (the amounts of which were determined by the experimental design), prior to aerosolization into a 500 mL beaker containing 6% *w*/*v* crosslinker TPP [13]. Before use, the CHT was dissolved in acetic acid (1% *w*/*v*), with the EUD dispersed in distilled water at room temperature using a magnetic stirrer (300 rpm). Aerosolization was achieved by spraying the prepared polymeric solution through a fluid bed drier nozzle (Mini Lab Coater, Umang Pharmatech, Maharashtra, India) at a constant rate of 5 mL/min, a nozzle height of 20 cm above the collection vessel and a 0.1 MPa air pressure. After aerosolization, the prepared microparticles were left at room temperature for 30 min prior to collection through centrifugation. The microparticles were thereafter washed with distilled water and lyophilized at −60 °C for 24 h at 25 mmtor. 

#### 2.2.1. In Silico Analysis of the Ciprofloxacin-Polymer Interactions

The Materials Science platform of Schrödinger software (version 2018-2) was used to assess the molecular interactions of ciprofloxacin and the CHT-EUD polymers. The 3D structure of the CHT was retrieved from Mol-Instincts (CT1078683894). This was then cross-linked with the Eudragit RL using a platform that allows for the formation of 3D structures, a 3D Builder tool; the complex was then subsequently refined by the protein preparation wizard [20,21]. In order to create a final CHT-EUD polymer, the prepared complex was submitted to the Polymer Builder tool with the standard settings, including the generation of an amorphous polymer under OPLS3e forcefield at a cut-off temperature of 300 K for the Boltzmann constant, the van der Waals clash scale factor of 0.5 and a density of 0.5 g/cm^3^ [22,23]. To prepare the polymer for drug loading, it was minimized and turned into a sphere by the Nanoparticle Builder at a standard cut-off radius of 5 Å. Next, to determine the interactions of the ciprofloxacin with this polymeric carrier system, the disordered structure of the CHT-EUD nanosphere was generated and ciprofloxacin was immersed as a substrate using a rigorous Disordered System Builder under the OPLS3e forcefield. The final complex was then assessed for crystal pose and intermolecular interactions [24,25,26].

#### 2.2.2. Construction of the Experimental Design

A face-centered central composite experimental design was utilized to ascertain the optimal formulation for the delivery of ciprofloxacin over 12 h. For the experimental design Chitosan (0.5–1.5% *w*/*v*) and Eudragit^®^ RL100 30D (0.5–2% *w*/*v*) were selected as the independent formulation variables with a Mean Dissolution at 12 h (MD_12_), drug entrapment efficiency (DEE) and microparticle yield (MPY) selected as the design responses. The experimental analyses were performed on statistically derived formulations composed of various combinations of CHT and EUD as highlighted in Table 1. All formulations were prepared at room temperature, as previously described. A statistical model incorporating interactive and polynomial terms was utilized to evaluate the responses. 

#### 2.2.3. Determination of the MPY and DEE

The MPY was determined by comparing the actual particle yield weight achieved after synthesis to the theoretical yield from the individual formulation components (Equation (1)) with the DEE calculated by dissolving microparticles (50 mg) in simulated human gastric fluid (SHGF) (100 mL; pH 1.2; 37 °C) for 24 h and determining the ciprofloxacin composition through UV spectroscopy (CE 3021, Cecil Instruments, Cambridge, UK) at 280 nm (Equation (2)):

(1) $$MPY=\;\frac{Actual\;Amount\;of\;Microparticles}{Theoretical\;Amount\;of\;Microparticles}\times 100$$

(2) $$DEE=\;\frac{Amount\;of\;Encapsulated\;Ciprofloxacin}{Theoretical\;Amount\;of\;Ciprofloxacin}\times 100$$

#### 2.2.4. In Vitro Drug Release Analysis

The in vitro drug release analysis of the ciprofloxacin-loaded microparticles (*n* = 3) was conducted employing a USP II dissolution apparatus (Erweka DT 700, Heusenstamm, Germany) set at 50 rpm. For the analysis, 50 mg of microparticles were placed in 200 mL SHGF. The samples (5 mL) were extracted at predetermined time intervals, filtered and analyzed by UV spectroscopy. An equal volume of fresh SHGF was replaced after each sample extraction. Fractional Drug Release (FDR) and MD_12_ for each design formulation was thereafter calculated. The FDR was calculated using Equation (3):

(3) $$FDR=\;\frac{Amount\;of\;Ciprofloxacin\;Released}{Amount\;of\;Total\;Dosed\;Ciprofloxacin}$$

#### 2.2.5. Constraint Optimization of the Formulation Responses

A model-independent approach (Minitab^®^ V15, Minitab Inc., State College, PA, USA) was used to optimize the ciprofloxacin-loaded microparticles for a maximum DEE and MPY, and an MD_12_ value of 34.833, conforming to zero-order kinetics over 12 h. The optimized system was formulated and analyzed for its DEE, MPY and MD_12_ values as previously described. Dissolution modelling on the optimized microparticle system was additionally undertaken using the Zero Order, First Order, Higuchi, Hixson–Crowell and Korsmeyer–Peppas models [27,28].

### 2.3. Preparation of the MGDDS Platform

The optimized lyophilized microparticles, containing 250 mg of ciprofloxacin were uniformly dispersed and crosslinked in a homogenous polymeric gastrosphere solution (composed of a mixture of 1% *w*/*v* ALG, 1% *w*/*v* PEC, 2% *w*/*v* PAA and 2% *w*/*v* PLGA) using calcium hydroxide (2% *w*/*v*) as the crosslinking solution. The prepared drug-loaded microparticle-entrapped gastrospheres (MGDDS) were thereafter cured for a further 30 min prior to filtering, collection, washing and lyophilization, as previously described.

#### 2.3.1. Characterization of the MGDDS

##### Surface Morphology

The SEM analysis was carried out on the optimized microparticles and the MGDDS using a Phenom™ scanning electron microscope (FEI Company, Hillsboro, OR, USA). Prior to the analysis, the samples were gold-sputter coated (SPI Module™ Sputter Coater, SPI Supplies, West Chester, PA, USA) for 90 s (18 mA).

##### Fourier Transmission Infrared Spectroscopy

The structural characterization of the prepared drug-free microparticles, gastrospheres and their native polymers was undertaken using a Spectrum 2000 FTIR spectrometer with a MIRTGS detector (PerkinElmer Spectrum 100, Beaconsfield, UK) to determine the properties of the cross-linked microparticles, as well as the chemical nature of the polymers used prior to and after the formulation process. All samples were analyzed at a resolution of 4 cm^−1^ for 16 scans over wave numbers from 4000–400 cm^−1^.

##### Swelling Potential

The calculation of the swelling potential of the prepared gastrospheres was achieved by immersing pre-weighed 50 gastrospheres in 100 mL SHGF. The solution was thereafter placed in an orbital shaker incubator rotating at 35 rpm at 37 °C for 12 h. The gastrospheres were thereafter blotted with filter paper to remove any excess SHGF and weighed. The swelling of the gastrospheres was calculated using Equation (4) [29]:

(4) $$Swelling=\;\frac{Hydrated\;Mass}{Dry\;mass}\times 100$$

### 2.4. In Vivo Analysis

Healthy female Large White pigs (35 ± 0.5 kg; *n* = 5) were used for the in vivo evaluation of the ciprofloxacin-loaded gastrospheres and the comparator 250 mg immediate-release marketed product. The study was divided into two groups with Group 1 administered with the ciprofloxacin-loaded gastrospheres (containing 250 mg ciprofloxacin in a hard gelatin capsule) and Group 2 with the marketed ciprofloxacin product via a gastric tube. The marketed product is routinely administered as a twice daily dose. A washout period of one day was maintained between dosing, with blood samples (10 mL) removed via a surgically implanted intra-jugular catheter, at 0, 2, 4, 6, 8, 10, 12, 16, 20 and 24 h after administration of the respective system. 

All blood samples were analyzed using the method by Pearce et al. [30] with modifications. Briefly, a Waters Acquity^®^ UPLC system (Waters Corp., Milford, MA, USA) equipped with an Acquity^®^ UPLC BEH shield Reverse Phase C18 column (2.1 mm × 100 mm I.D. 1.7 µm) set at 25 °C, a detection wavelength of 280 nm, a 5 min run time and a 5 µL injection volume was used. Prior to the analysis, plasma (1 mL) was precipitated with acetonitrile (0.2 mL), diluted with deionized water, and centrifuged (3200 rpm) for 15 min. The supernatant was thereafter removed and injected into a Waters Oasis HLB 3cc cartridge conditioned with deionized water (1 mL) and methanol (1 mL). The samples were thereafter washed with deionized water (1 mL) and 10% methanol (1 mL) with ciprofloxacin eluted with 1 mL of acetonitrile:ammonia/ammonium buffer (1:1). The mobile phase consisted of both the buffer and acetonitrile under gradient conditions (0.0 min: 90:10 (Buffer:ACN); 0.5 min: 80:20; 3.0 min: 90:10) with ranitidine used as the internal standard.

#### Pharmacokinetic Modelling

Pharmacokinetic modelling was achieved using PKSolver to undertake a non-compartmental and compartmental analysis of the in vivo plasma ciprofloxacin levels and thus determine the AUC and plasma half-life of ciprofloxacin from the MGDDS and commercial comparator product [31].

## 3. Results

### 3.1. Analysis of the Ciprofloxacin Interactions with CHT-EUD Polymer through Molecular Modelling

**Figure 2:**
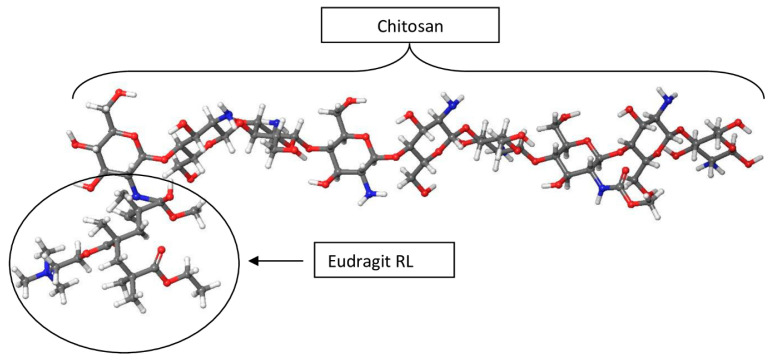
The CHT-EUD nanosphere displaying the EUD head and CHT tail.

The CHT-EUD polymer virtual synthesis using molecular modelling technology was initiated on a 3D workspace known as a 3D Builder. The CHT and EUD were crosslinked by the 3D Builder tool to form a co-polymer that was further polymerized under the Polymer Builder panel. The Nanoparticle Builder subsequently generated a nanosphere from the final polymer showing the positively charged Eudragit RL head and the CHT tail (Figure 2) in agreement with the literature [32,33].

**Figure 3:**
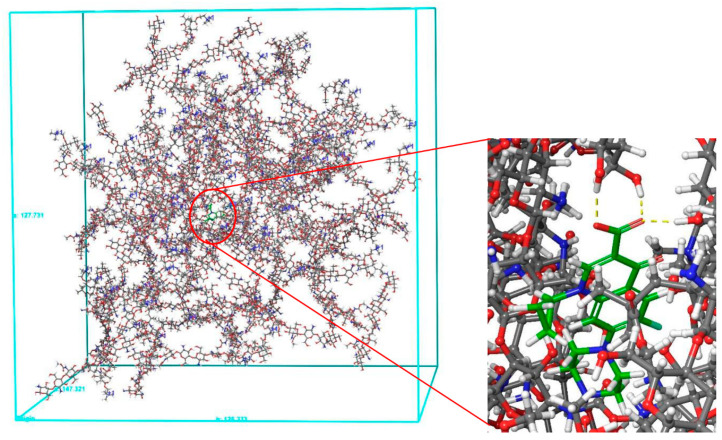
Disordered system of the ciprofloxacin (green) loaded CHT-EUD carrier system.

The central composite of the ciprofloxacin-loaded CHT-EUD carrier system was generated from the Disordered System Builder using the nanospheres with ciprofloxacin immersed as a substrate. This showed favorable interactions of ciprofloxacin with the CHT-EUD carrier system where the terminal carboxyl group of the drug formed hydrogen bond interactions with the hydroxyl groups of the carrier polymer (Figure 3).

**Figure 4:**
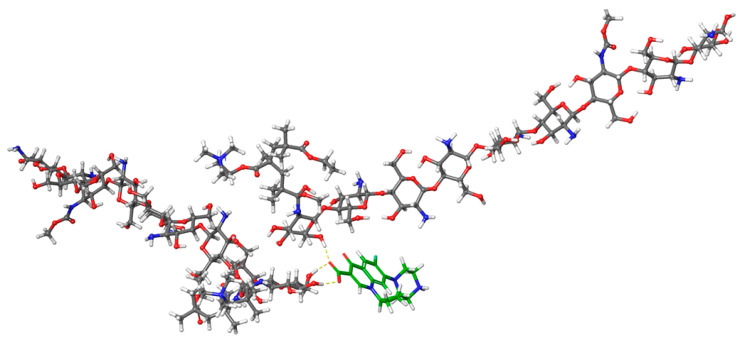
Detailed analysis of ciprofloxacin and CHT-EUD regions involved in hydrogen bonding.

To further identify the polymer interaction site as well as the polymer configuration in the composite, the two monomers involved in the drug interaction were selected for analysis (Figure 4). This revealed that CHT-EUD interacted with the ciprofloxacin at the crosslink of the co-polymer mainly with the terminal glucosamine moiety of the CHT. Interestingly, this interaction pulled the crosslinking portion inward which then provided for the outward facing of the EUD, exposing the positively charged amine groups to the surface. This is the best configuration since the positively charged EUD is known to promote the cellular uptake of the drug through the ionic interactions with the negatively charged cellular membranes [34,35]. Moreover, the rest of the ciprofloxacin structure interacted with the polymers through the weak van der Waals forces. The balanced hydrogen bonding with the weaker van der Waals forces provide for the controlled release properties of the drugs [35], suggesting that the ciprofloxacin release will be sufficiently controlled.

### 3.2. Analysis of the Central Composite Experimental Design

**Figure 5:**
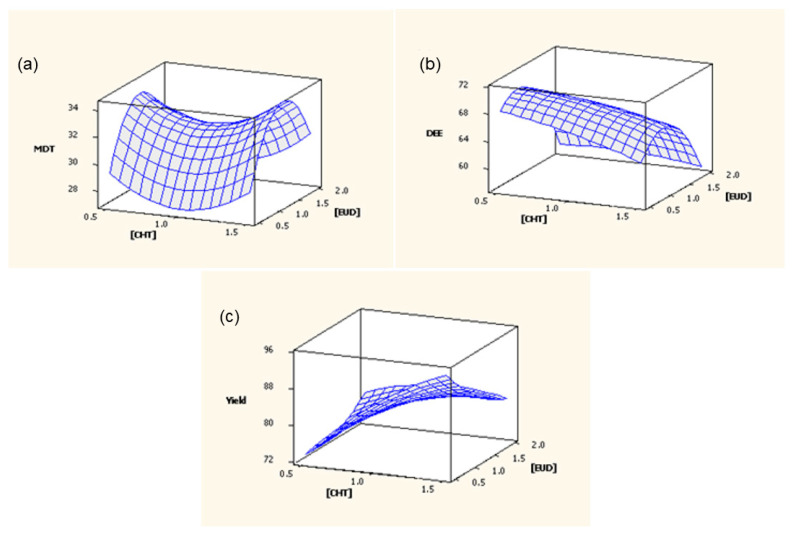
Response surface plots depicting the effect of polymer concentrations on (**a**) MD_12_, (**b**) DEE and (**c**) MPY.

The MD_12_ values (as displayed in Table 2) noted values for the respective formulation of between 25.36 and 34.91 h, displaying the varying potential of the microparticle system to control the release of ciprofloxacin. It was also noted from these results that the EUD composition in the microparticle system appeared to have a greater influence on the in vitro ciprofloxacin release when compared to CHT. This was further depicted in the response surface plot (Figure 5a), where the respective trend in the MD_12_ values, in response to the varying CHT and EUD concentrations, is depicted. The highest MD_12_ was realized at a moderate EUD concentration (1.25% *w*/*v*) and decreased in the higher or lower ranges. Similarly, MD_12_ initially increased with an increase in the CHT concentration; however, this trend was reversed when CHT reached 2.0%. These results were attributed to the microparticles not being able to swell enough at the lower ranges of EUD to permit the passage of the drug through the pores, while at the higher concentrations the swelling capability was inhibited or restricted by the rigid crosslinked CHT structure. It was also noted that for any given amount of EUD and TPP, an increase in the CHT concentration resulted in an increased MD_12_. This could also be attributed to the enhanced complexation of CHT with EUD and the increased intermolecular crosslinks with TPP.

The DEE was determined to be within a range of 50% to 73% for all the design formulations (Table 2). The evaluation of the influence of CHT and EUD on DEE (as provided in the response surface plot in Figure 5b) noted that at lower CHT concentrations, DEE was higher. Additionally, at lower concentrations of EUD (0.5 to 1% *w*/*v*) and at an increased concentration (2% *w*/*v*), a decreased DEE was seen. A similar trend was also noted at high concentrations of CHT. This result was attributed to a loose network at low concentrations of EUD, potentially due to drug leaching during the formulation process. Furthermore, at higher EUD concentrations, the polymer network formed may become too dense, inhibiting drug entrapment. 

The MPY for the design formulations was relatively high (77 to 92%), with only one formulation (Formulation 13) deviating from this range at 65% (Table 2). The influence of CHT and EUD on MPY is depicted in Figure 5c, where at low CHT concentrations MPY was low, with a higher MPY at increased CHT concentrations. Additionally, an increase in the EUD composition resulted in a decrease in MPY. The decreased MPY can be explained by the increase in viscosity of the polymer solution because of the increased CHT concentration, with a viscous solution more resistant to fragmentation into small droplets.

#### Response Optimization

The response optimization, targeting an MD_12_ value of 34.883, a maximum DEE and MPY indicated that the optimized formulation was obtained at 1.5% CHT and 1.0741% EUD. The optimized formulations were determined to have a desirability of >0.9 with the actual vs. predicted responses being ≥90%.

### 3.3. Characterization of the Optimized Microparticle-Loaded Gastrosphere

#### 3.3.1. Surface Morphology Characterization 

**Figure 6:**
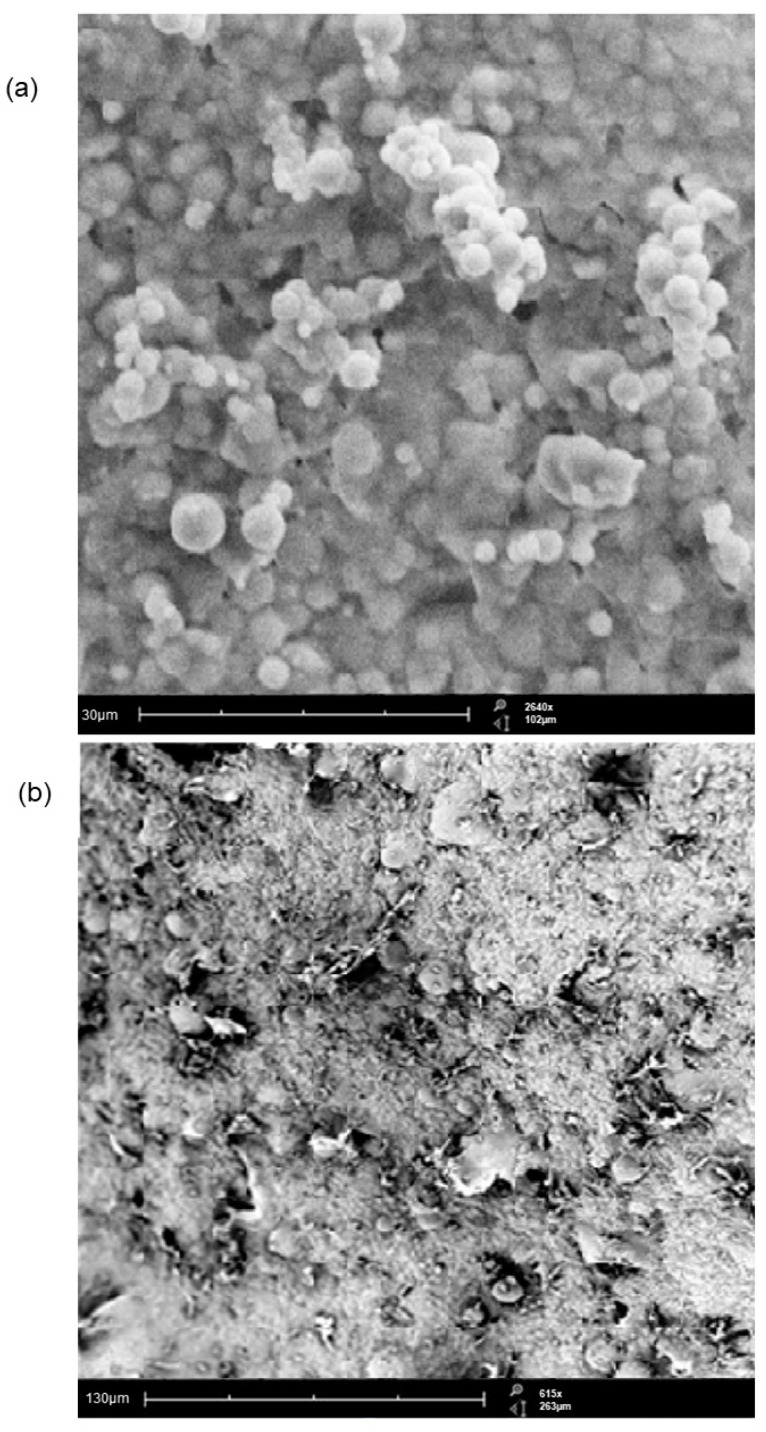
SEM images of (**a**) the optimized microparticle system (magnification: 2640×) and (**b**) MGDDS (magnification: 615×). Image reproduced with permission from reference [36], © University of the Witwatersrand, South Africa.

SEM imaging of the optimized microparticles and the MGDDS (as depicted in Figure 6) was performed to determine the size of the prepared microparticles, as well as the surface characteristics of the microparticles and the MGDDS. The evaluation of the optimized microparticles (Figure 6a) determined that consistently smooth-edged spherical particulates of between 1–4 µm were formed with no distinct surface pores. Additionally, for the MGDDS SEM image (Figure 6b), air bubbles or voids within the gastrosphere structure were seen, with embedded microparticles visible on the surface. The porous nature of the surface of the gastrospheres is essential for the hydration of the system to allow for drug release, while the air bubbles provide the buoyancy expected of the gastro-retentive systems and are required for their functionality. The voids seen were due to the lyophilization process, whereby water crystals were removed from the system through sublimation. The surface morphology of the gastrosphere also detailed a solid surface with numerous pores, correlating with the data seen in the experimental design.

#### 3.3.2. FTIR Analysis of MGDDS

**Figure 7:**
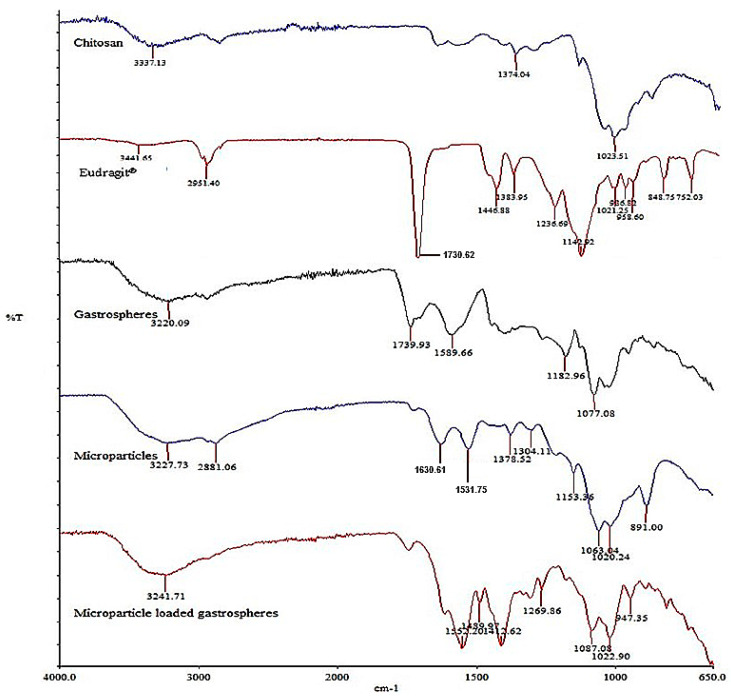
FTIR spectra of a drug-free microparticle, the gastrosphere system (MGDDS) and its native components.

The evaluation of the FTIR spectra of the microparticle, the gastrosphere and its component polymers (as provided in Figure 7) revealed that the NH stretching vibrations (at wavenumbers 3300–3500 cm^−1^) were found in the native CHT and EUD compounds, which were still noted in the prepared microparticles [37]. Additionally, C=O vibrations were seen in the native EUD spectra (between wavenumbers 1700 and 1900 cm^−1^) and again in the evaluation of the microparticle formulation; however, with a decreased intensity [38]. The other C-H bands (at wavenumbers 2800–2950 cm^−1^; 1355–1395 cm^−1^; 1405–1465 cm^−1^ and 1430–1470 cm^−1^) were noted to be present in all the spectra. The C=O ester (at the 1730 cm^−1^ peak) seen in the native EUD spectra, was, however, significantly diminished in the microparticle spectra with a new peak formed at 1531 cm^−1^. This result is consistent with the previous research on polymer systems consisting of CHT and EUD [39,40]. The noted diminishing of the esterified carboxyl group bands can also be attributed to the amount of CHT present, with the CHT concentration in the microparticle system being greater than the EUD [40]. It was also noted that the strong bands at ±3200 cm^−1^ observed for both the microparticle and gastrosphere systems, were still present after the formulation of the MGDDS. The results of this analysis therefore confirmed that the chemical nature of the native polymers was maintained during the production process and that there was no chemical interaction due to the incorporation of the microparticles into the gastrosphere system. This result is of importance as changes to the chemical structure can affect the functionality and safety of the polymer system.

#### 3.3.3. In Vitro Drug Release

**Figure 8:**
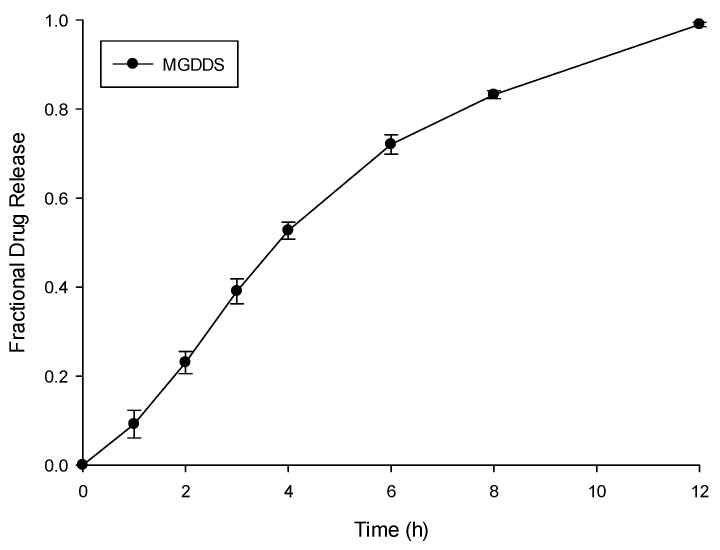
The ciprofloxacin release profile of MGDDS in SHGF (pH 1.2, 37 °C; *n* = 3; SD ≤ 0.031 in all cases).

The ciprofloxacin release profile from the MGDDS (Figure 8), which shows the FDR of ciprofloxacin over the 12 h test period, depicted the controlled release of drug with a 0.092 FDR (9.2%) achieved after 1 h, a 0.23 FDR (23.0%) after 2 h, rising to a 0.52 FDR (52.7%) after 4 h. While controlled in nature, this result can be attributed to the initial rate of swelling allowing for a rapid drug release. The rate of release then begins to decrease with an FDR of 0.832 (83.2%) after 8 h with a final FDR of 0.99 (99.0%) achieved after 12 h. This result can be attributed to the decreased amount of drug present in the matrix after 6 h, causing a decrease in the rate of release. The analysis of the swelling potential of the prepared gastrospheres detailed a 445% (±32%) increase in mass after exposure to SHGF. This substantial increase in mass due to hydration is in correlation with the in vitro release data which reflected an initial rapid release prior to an FDR of 0.99 achieved after 12 h. The dissolution modeling of the release data, as provided in Table 3, further revealed that the optimized microparticle-loaded gastrosphere system best conformed to the Hixson–Crowell model (R = 0.988); however, a good linearity was also achieved when modelled with the Korsmeyer–Peppas model (R = 0.971; N value = 0.716). Through the use of these models, it can be conferred that a uniformity in release was achieved through hydration of the prepared gastrosphere system and that diffusion was a primary mechanism of release from the MGDDS. 

### 3.4. In Vivo Analysis of MGDDS

**Figure 9:**
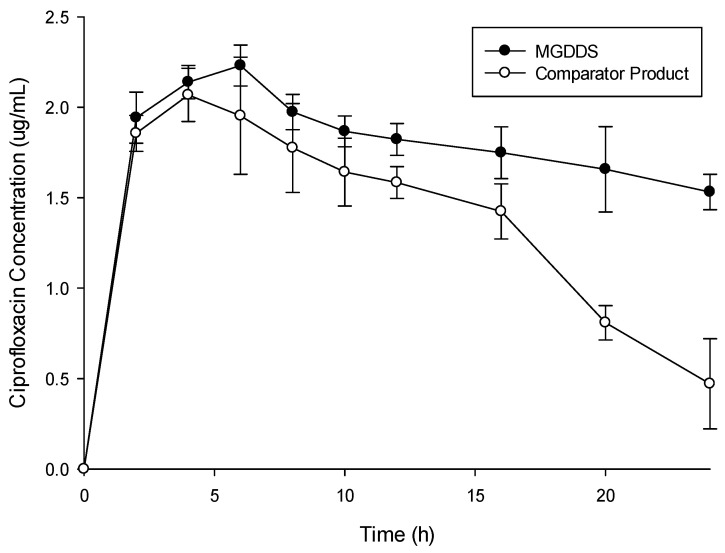
In vivo ciprofloxacin release from the microparticle loaded gastrospheres (*n* = 5, SD < 0.236 in all cases) and the commercial comparator product (*n* = 5, SD < 0.324 in all cases).

The in vivo drug release profiles of the MGDDS and the commercial comparator product (as depicted in Figure 9) displayed a high plasma level of ciprofloxacin from the MGDDS within a shorter period rising to a higher C_max_ (2.25 µg/mL at T_max_ 5.08 h) when compared to the commercial product (C_max_ of 2.0 µg/mL at T_max_ 4.0 h). The 12.5% increase in C_max_ noted can also be attributed to the enhanced absorption from the MGDDS. This increase however may be potentially substantial when delivering highly potent drugs, which will require further optimization of the MGDDS system. Additionally, the AUC_0–12_ of the MGDDS system was calculated to be 42.46 μg.h/mL while the commercial product had an AUC_0–12_ of 33.21 μg.h/mL. This detailed the greater residence time of ciprofloxacin within the cardiovascular system in addition to a greater bioavailability. It was also determined that the drug release was sustained, and the absorption controlled from the MGDDS, while with the commercial product, the ciprofloxacin concentrations decreased steadily after reaching the peak concentration. This was again seen upon the compartmental analysis and calculation of the t_1/2_, which for the gastrosphere system was 37.70 h with the commercial product having a t_1/2_ of 11.39 h. Overall, these results indicate that the MGDDS platform portrayed a superior bioavailability and drug residence when compared to the commercial product.

## 4. Discussion

The NAW drugs are known for their erratic and often decreased absorption profiles due to the limited capacity of the intestinal tract to absorb these molecules. Through the use of gastrofloatable systems, that conform to preferred densities, sizes and shapes, the controlled release of a drug can be achieved to allow for a more effective absorption process, overcoming this biopharmaceutical concern. Gastrofloatable systems, or formulations with a lower density than gastric fluid are the most practical and researched platforms for the delivery of NAW drugs due to their predictability and uncomplicated formulation processes [41]. Gastrofloatable systems are categorized into two subtypes of platforms: effervescent and non-effervescent floating systems, with the non-effervescent system based on the utilization of highly swellable or gel-forming polymers to form hydrodynamically balanced systems (HBS). The use of HBS allows for a predictable release of loaded drugs through the appropriate control of the formulation matrix. This however is not always the case with the release kinetics of the dosed drug being dependent upon the floating properties of the system and vice versa [41,42]. In this study, it was shown that optimization of the polymer-based microparticles, which are thereafter incorporated in gastrofloatable gastrospheres can effectively control the delivery of NAW drugs, such as ciprofloxacin, enhancing its absorption when compared to a conventional immediate-release formulation. Previous research on gastroretentive systems for the delivery of the NAW drug metformin has also been undertaken using similar polymers with in vitro release data displaying a controlled release of the loaded drug in simulated gastrointestinal media [13,43]. 

During the optimization of the microparticles prepared in this study, which were composed of CHT and EUD, it was noted that a DEE of between 50.83% and 73.24% and Mean Dissolution Time values of 25.36 and 34.91 h was achieved over the various experimental design formulations, highlighting the potential of the microparticles to be statistically optimized to varying drug release profiles. This is of significance as other NAW drugs could potentially be included in microparticles comprising of CHT and EUD for a controlled release. The SEM imaging additionally displayed uniform microparticles of an ideal size and shape, as well as gastrospheres with a porous surface, which is a property required for adequate hydration in gastrofloatable systems and air bubbles and voids allowing for buoyancy in the gastric media. A simulation using molecular modelling additionally unveiled that the ciprofloxacin-loaded CHT-EUD assumed a conformation that placed the positive charge on the outer surface. This suggested an increased cellular uptake of ciprofloxacin and its subsequent bioavailability, resulting from the ionic interactions of the delivery system with the negatively charged cellular membranes [34,35]. Additionally, the mixed hydrogen bonding and hydrophobic interactions potentially contributed to the controlled release profile of the ciprofloxacin from the CHT-EUD-based delivery system.

The gastrosphere system, incorporating the optimized microparticle system, was further noted in vitro to release the ciprofloxacin in a controlled-release manner over the 12 h test period, with the in vivo studies displaying a higher C_max_ and a greater plasma concentration after 24 h when compared to an immediate-release comparator product. The future applications of the MGDDS would therefore be for the delivery of other NAW drugs and for a comparison to marketed controlled-release formulations. The results of this study however have shown the superior release and absorption profiles of ciprofloxacin when utilizing a micro-in-macro gastrofloatable system, highlighting the potential for further optimization and functionalization of such systems.

## 5. Conclusions

In this study, a novel microparticle-entrapped gastrosphere designed to deliver ciprofloxacin in a gastric-retentive manner was prepared, optimized and analyzed for its structural, morphological and drug release properties. The optimized system displayed a controlled in vitro ciprofloxacin release over 12 h with the in vivo results determining that the blood plasma concentrations of ciprofloxacin were much higher and remained more constant when compared to the marketed comparator product. Overall, the experimental findings proved the capacity of the developed platform to be used for the enhanced delivery of ciprofloxacin and potentially other NAW drugs with similar physicochemical properties that favor NAW profiles.

## Figures and Tables

**Table 1:** The Face-Centered Central Composite Experimental Design formulations.

Formulation	Chitosan (0.5–1.5%)	Eudragit^®^ RL 30D (0.5–2%)
1	1.5	2.00
2	0.5	2.00
3	1.0	1.25
4	1.0	1.25
5	0.5	0.50
6	1.5	0.50
7	1.0	1.25
8	1.0	2.00
9	1.5	1.25
10	1.0	1.25
11	1.0	1.25
12	1.0	1.25
13	0.5	1.25
14	1.0	0.50

**Table 2:** The MD_12_, DEE and MPY values for the ciprofloxacin-loaded design gastrosphere formulations (*n* = 3).

Formulation Number	MD_12_ (h) *	DEE (%) **	MPY (%) ***
1	29.78	50.83	77.08
2	29.79	57.23	82.43
3	32.24	69.61	83.49
4	32.07	64.45	85.45
5	30.82	71.28	79.02
6	29.48	60.57	92.19
7	32.4	67.55	79.66
8	29.33	64.70	83.57
9	34.91	73.24	92.25
10	31.87	68.75	81.55
11	32.10	66.45	81.24
12	32.00	69.67	82.85
13	32.47	65.91	65.84
14	25.36	64.23	83.19

* SD ≤ 0.738 in all cases, ** SD ≤ 5.616 in all cases, *** SD ≤ 7.442 in all cases.

**Table 3:** Dissolution modelling of the in vitro ciprofloxacin release data.

Dissolution Model	Parameter
Hixson–Crowell	R = 0.988 K_HC_ = 0.054
Zero Order	R = 0.907 K_0_ = 9.741
First Order	R = 0.968 K_1_ = 0.191
Higuchi	R = 0.920 K_H_ = 26.931
Korsmeyer–Peppas	R = 0.971 K_KP_ = 17.836 N = 0.716

## Data Availability

The data emanating from this study are available on request from the corresponding author.
